# Antibiotic Resistance of* Campylobacter* Recovered from Faeces and Carcasses of Healthy Livestock

**DOI:** 10.1155/2017/4091856

**Published:** 2017-01-18

**Authors:** Akosua B. Karikari, Kwasi Obiri-Danso, Enoch H. Frimpong, Karen A. Krogfelt

**Affiliations:** ^1^Department of Clinical Microbiology, University for Development Studies, Tamale, Ghana; ^2^Department of Theoretical and Applied Biology, Kwame Nkrumah University of Science and Technology, Kumasi, Ghana; ^3^Department of Clinical Microbiology, Kwame Nkrumah University of Science and Technology, Kumasi, Ghana; ^4^Department of Microbiology & Infection Control, Statens Serum Institute, Copenhagen, Denmark

## Abstract

*Campylobacter* is of major significance in food safety and human and veterinary medicine. This study highlighted resistance situation in the area of veterinary public health in Ghana. Using selective mCCDA agar, isolates were confirmed phenotypically on API CAMPY and genotypically by multiplex PCR of* IpxA* gene. The susceptibility profile of species to common and relevant antibiotics was determined by the Kirby-Bauer disk diffusion method. Cattle, sheep, goat, and pig faecal samples analysed, respectively, yielded 13.2% (16/121), 18.6% (22/102), 18.5% (25/135), and 28.7% (29/101)* Campylobacter* species while 34.5% (38/110), 35.9% (42/117), 23.9% (32/134), and 36.3% (37/102) were, respectively, recovered from the carcasses. Species identified in faeces were* C. jejuni* 35.8% (33/92),* C. jejuni* subsp.* doylei* 4.3% (4/92),* C. coli* 47.8% (44/92), and* C. lari* 12.0% (11/92). Species discovered in carcasses were* C. jejuni* 83.9% (125/149),* C. jejuni* subsp.* doylei* 2.0% (3/149),* C. coli* 6.0% (9/149), and* C. lari* 8.1% (12/149). Resistance ranged from 92 to 97% to the *β*-lactams, 7 to 69% to the quinolones, 0 to 44% to the aminoglycosides, 97 to 100% to erythromycin, 48 to 94% to tetracycline, 45 to 88% to chloramphenicol, and 42 to 86% to trimethoprim/sulfamethoxazole as 0% resistance was observed against imipenem.

## 1. Introduction

*Campylobacter* is a key zoonotic pathogen which causes foodborne enteritis with* C. jejuni* and* C. coli* being the most isolated species [1].* Campylobacter* infections in humans are mainly associated with consumption of undercooked chicken as exposure to farm animals, consumption of pork, improperly cooked beef, raw milk, and untreated water are other transmission routes. The significant contribution of ruminants as important reservoirs of* Campylobacter *has been established through molecular epidemiological research [2]. Due to growing demand for meat and products of livestock, the possibility of disease transmission from these food animals' sources cannot be dismissed. Increasing antibiotic resistance in* Campylobacter* from animal sources is well reported globally [3–6]. Resistance has developed to nearly all antibiotics used in veterinary medicine [7].

Livestock production in Ghana currently is changing from free range to commercial productions with increased use of antimicrobials as growth promoters and therapeutic agents. Such practice could increase levels of resistant bacteria in the gut-intestinal flora of animals, such as* Campylobacter*, and subsequently increase resistance in foods due to faecal contamination during slaughter. In Ghana, regional reports of heightening drug resistance among several pathogens from human sources have been documented [8]; however research on resistance of bacteria from animal sources is very sketchy. The purpose of this study was to highlight resistance trends in the area of veterinary public health. Therefore isolation rate and level of antimicrobial resistance among* Campylobacter* species from faeces and carcasses of livestock were reported.

## 2. Materials and Method

### 2.1. Study Site

The study was conducted at the Kumasi Abattoir. Kumasi is the capital of Ashanti region and the second most populous Metropolis in Ghana. The abattoir supplies Ghanaian markets with slaughtered, processed, and packaged goats, cattle, pigs, and sheep. The daily slaughtering capacity is about 200 cattle, 100 pigs, and 250 sheep and goats. Animals intended for slaughter at the abattoir are transported from different regions within Ghana especially from Yeji in Brong Ahafo and the Northern regions and also from neighbouring countries like Burkina Faso, Mali, and Niger.

### 2.2. Sample Collection

Fresh faecal samples were collected from individual animals (cattle, pigs, goats, and sheep) from unrelated herds at the Kumasi Abattoir slaughterhouse complex. A single animal was selected at random and about 5 g of faeces was aseptically removed from the bowel after evisceration at the slaughtering line. Samples were collected into sterile ziplock bags, kept on ice and returned to the laboratory for processing. Carcasses of cows, sheep, goats, and pigs being packaged at the abattoir for the markets were randomly swabbed using sterile swab sticks and inoculated into sterile Amies Transport Medium (Copan ESwab sticks, Italy) and transferred to the laboratory on ice. Sampling took place from March 2013 to February 2014.

### 2.3. Processing, Isolation, and Identification

About 0.2 g of faecal material was plated directly onto mCCDA agar plates (Oxoid CM0698) using sterile swab stick and incubated at 42°C for 48 hrs. Carcass swabs together with the transport media were aseptically transferred into sterile bijou bottles and preenriched with 5 mL of blood-free* Campylobacter* broth (Oxoid CM0963) supplemented with CCDA selective supplement (Oxoid, SRO 155E) and incubated overnight at 37°C. The overnight enrichment culture was cultured onto mCCDA agar plate and incubated at 42°C for 48 hrs. CampyGen (Oxoid CN0025A) was introduced to provide microaerophilic condition. Colonies showing typical morphology of* Campylobacter *spp. were subcultured onto Nutrient agar, followed by biochemical tests including Gram stain, oxidase, and catalase. Isolates which were small, curved, catalase, and oxidase positive; Gram negative bacilli were presumed to be* Campylobacter* spp. The presumed isolates were further subjected to standard phenotypic tests using API CAMPY system (bioMérieux, Marcy l'Etoile, France) to identify to species level.

### 2.4. Genotyping of* Campylobacter* Species by Multiplex PCR

#### 2.4.1. DNA Extraction

Genomic DNA was isolated from cultures grown on 5% sheep blood agar (Accumix, AM5014, India) for 24 to 48 h at 37°C under microaerophilic conditions. Cell lysates were prepared by suspending a 10 *μ*L loopful of growth in 100 *μ*L of sterile distilled water in a microcentrifuge tube. The tubes were heated at 100°C for 10 min and subsequently cooled to 4°C. The tubes were centrifuged at 13,000 rpm (Sigma, Germany) for 5 min, and the supernatant was stored at −20°C for further analysis.

#### 2.4.2. Genus-Specific PCR Amplification

The primers of the* lpxA* gene (DNA Technology, Denmark) were used in this study [9]. Forward primers complementary to the* lpxA* nucleotide sequence of* C. coli *(*lpxA C. coli*),* C. jejuni* (*lpxA C. jejuni*),* C. lari* (*lpxA C. lari*), and* C. upsaliensis* (*lpxA C*.* upsaliensis*) were used in combination with the reverse primer* lpxARKK2m* for confirmation of* Campylobacter *species by multiplex PCR with expected amplicon (fragment) sizes (Table 1). The reaction mixture consisted of 2 *μ*L Taq buffer 10x, 0.7 *μ*L Taq polymerase (Fermentas, UK), forward primer 50 pmol (0.5 *μ*L), 50 pmol (0.5 *μ*L) reverse primer, dNTPs 5 Mm, 0.4 *μ*L, and nuclease free water 12.9 *μ*L, 0.5 *μ*L of each primer, and 1.5 *μ*L of the genomic DNA template. The reaction tubes (20 *μ*L) were placed in the thermal cycler (Thermo PCR, Sprint, USA). The cycle involved initial denaturing at 94°C for 5 min. The denatured DNA was maintained at 94°C for 1 min followed by annealing of primers to the DNA template at 50°C for 1 min; the annealed primers were extended at 72°C for 2 min. This cycle was repeated 30x and a final filling-in at 72°C was carried out for 10 min [9]. The PCR products were run on 1% agarose gel (Sigma, Germany) at 100 V for 45 minutes; the separated PCR products (bands) were visualized with UV imager (Syngene, USA). A 1 kb ladder was used as a molecular size standard.* Campylobacter* strains obtained from Statens Serum Institute (Denmark) were used as positive control and a negative control was included and examined.

### 2.5. Antibiotic Susceptibility Test

Susceptibility tests were performed by the Kirby-Bauer disk diffusion method on Mueller-Hinton agar (Liofilchem, Italy) supplemented with 5% sheep blood following CLSI guidelines. Antibiotics tested and their corresponding concentrations were ampicillin (10 *μ*g/disc), chloramphenicol (30 *μ*g/disc), ciprofloxacin (5 *μ*g/disc), kanamycin (30 *μ*g/disc), erythromycin (15 *μ*g/disc), gentamicin (10 *μ*g/disc), nalidixic acid (30 *μ*g/disc), tetracycline (30 *μ*g/disc), cephalexin (30 *μ*g/disc), trimethoprim/sulfamethoxazole (25 *μ*g/disc), norfloxacin (10 *μ*g/disc), cefotaxime (30 *μ*g/disc), and imipenem (10 *μ*g/disc). Mueller-Hinton agar plates were inoculated with 0.5 McFarland suspension and incubated under microaerophilic condition at 48°C for 24 hours. The inhibition zones were recorded and interpreted following EUCAST and CLSI breakpoints for* Campylobacter*. Breakpoints established by EUCAST and CLSI 2013 for Enterobacteriaceae were used to interpret the results of norfloxacin, trimethoprim/sulfamethoxazole, cefotaxime, and kanamycin as CLSI* Campylobacter* breakpoints for these antibiotics have not yet been established. Quality control was achieved by* Escherichia coli* (ATCC 25922) and* Staphylococcus aureus* (ATCC 25923) strains.

### 2.6. Statistical Analysis

Data were transferred to a Microsoft Excel spreadsheet for analysis. Descriptive analysis was carried out using percentages. Associations were determined using the Chi-square test at a significance level of < 0.05. All statistical tests were two-tailed. Stata 14.0 software was used for statistical analysis.

## 3. Results

### 3.1. Prevalence of* Campylobacter* in Food Animals

Of the cattle, sheep, goat, and pig faecal samples, 16 (13.2%), 22 (18.6%), 25 (18.5%), and 29 (28.7%) were, respectively, characterized as* Campylobacter* species. From the carcasses of the cattle, sheep, goats, and pigs, 34.5% (38/110), 35.9% (42/117), 23.9% (32/134), and 36.3% (37/102) isolates were positive for* Campylobacter *(Table 2). There was statistically significant difference in the isolation rate of* Campylobacter* from faecal and carcass samples of cattle (*p* < 0.001) and sheep (*p* = 0.003) but no significant difference in the isolation frequency between that of goats (*p* = 0.268) and pigs (*p* = 0.250) (Table 3).

### 3.2. Distribution of* Campylobacter* Species in the Various Animals

Faecal content isolates of cattle, sheep, goat, and pigs were 25.0%, 27.2%, 36.0%, and 48.2%* C. jejuni,* 43.7%, 40.9%, 56.0%, and 48.2%* C. coli,* and 12.5%, 27.2%, 8.0%, and 3.4%* C. lari, *respectively*. Campylobacter *subsp.* doylei *were recovered from cattle (18.7%) and sheep (4.5%) and none in goats and pigs. The carcasses of cattle, sheep, goats, and pigs were 84.2%, 92.8%, 81.3%, and 75.6%* C. jejuni* and 2.6%, 2.3%, 18.7%, and 10.8%* C. lari*.* Campylobacter coli* were found in cattle (13.1%) and pigs (10.8%) but none in sheep and goats, while* C. *subsp*. doylei* were obtained from sheep (4.7%) and pigs (2.7%) but not in goat and cattle samples (Table 4).

### 3.3. Molecular Identification and Confirmation of Isolates

**Figure 1:**
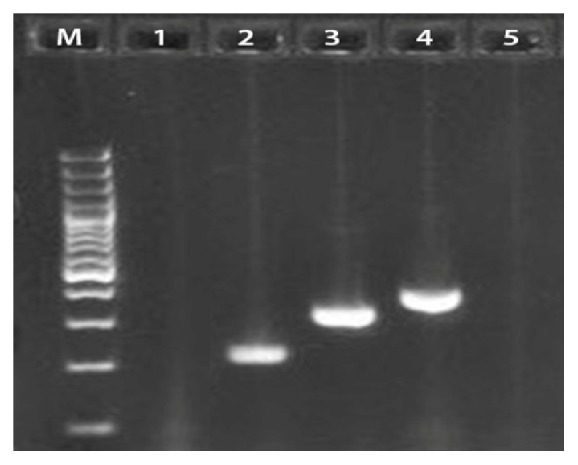
Multiplex PCR detection of* Campylobacter lpxA* gene on agarose gel electrophoresis. M = Molecular Marker 1 kb; Lane 2 =* C. lari* (233 bp); Lane 3 =* C. jejuni* (331 bp); Lane 4 =* C. coli* (391 bp); Lanes 1 and 5 = negative control.

**Figure 2:**
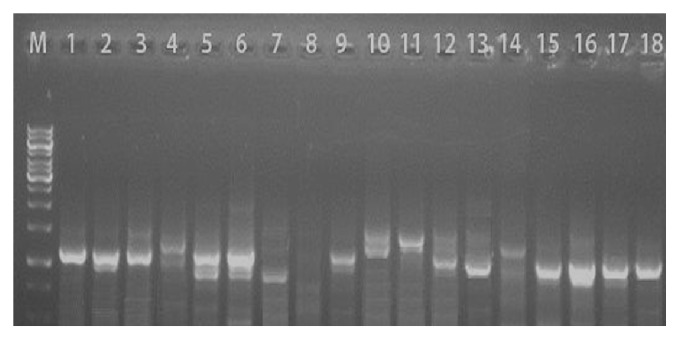
Multiplex PCR detection of* Campylobacter lpxA* gene of isolates on agarose gel electrophoresis. M = Molecular Marker 1 kb; Lanes 1, 2, 3 5, 6, 15, 16, 17, and 18 =* C. jejuni*; Lanes 4, 10, 11, and 14 =* C. coli*; Lane 7 =* C. lari*.

The PCR of the (*lpxA*) gene of* Campylobacter* confirmed 92 out of 96 faecal and carcass isolates from the various food animals. Multiplex PCR identified 52/92 (56.5%%) as* Campylobacter jejuni*, 33/92 (35.8%) as* Campylobacter coli*, and 7/92 (7.6%) as* C. lari* (Figures 1 and 2). Culture method and API reactions were 95.8% in agreement with the PCR results.

### 3.4. Resistance Profile of Faecal Isolates of Animals

Cattle isolates' resistance to erythromycin, chloramphenicol, and trimethoprim/sulfamethoxazole was, respectively, 100%, 88%, 56%, and 81% each to ampicillin and tetracycline. Against the cephalosporins resistance was 75% to cefotaxime and 81% to cephalexin. Resistance to the quinolones was 50% to nalidixic acid, 44% to ciprofloxacin, and 19% to norfloxacin. Resistance to the aminoglycosides was 6% to kanamycin and 0% to gentamicin.

Sheep isolates' resistance was 100% to erythromycin, 86% to trimethoprim/sulfamethoxazole, 91% each to ampicillin and tetracycline, and 55% to chloramphenicol. Against the cephalosporins resistance was 86% to cefotaxime and 91% to cephalexin. Resistance to the quinolones was 50% to ciprofloxacin and 36% each to nalidixic acid and norfloxacin. Against the aminoglycosides, resistance was 18% to kanamycin and 14% to gentamicin.

Goat strains expressed resistance of 100% 88%, 76%, 64%, and 60%, respectively, to erythromycin, ampicillin, tetracycline, chloramphenicol, and trimethoprim/sulfamethoxazole. Against the cephalosporins resistance was 72% to cephalexin and 68% to cefotaxime. Resistance to the quinolones was 28% to ciprofloxacin, 12% to norfloxacin, and 8% to nalidixic acid. Against the aminoglycosides, resistance was 8% to kanamycin and 0% to gentamicin.

Pig isolates showed resistance of 100%, 86%, 83%, 48, and 45%, respectively, to erythromycin, tetracycline, ampicillin, trimethoprim/sulfamethoxazole, and chloramphenicol. Against the cephalosporins resistance was 86% to cephalexin and 62% to cefotaxime. Resistance to the quinolones was 24% to ciprofloxacin, 17% to norfloxacin, and 10% to nalidixic acid. Resistance to the aminoglycosides was 10% each to kanamycin and gentamicin (Table 5). No resistance (0%) was recorded against imipenem; however intermediate susceptibility was observed among strains from all the animals.

### 3.5. Resistance Profile of Carcass Isolates of Animals

Cattle isolates showed resistance of 97% each against erythromycin and ampicillin, 87%, 58%, and 42%, respectively, to chloramphenicol, tetracycline, and trimethoprim/sulfamethoxazole. Against the cephalosporins resistance was 97% to cephalexin and 92% to cefotaxime. Resistance to the quinolones was 42% to ciprofloxacin, 21% to norfloxacin, and 13% to nalidixic acid. Against aminoglycosides, resistance was 19% to kanamycin and 18% to gentamicin.

Sheep isolates showed resistance of 100%, 93%, 83%, 52%, and 48%, respectively, to erythromycin, ampicillin, chloramphenicol, trimethoprim/sulfamethoxazole, and tetracycline. Against the cephalosporins resistance was 100% to cefotaxime and 98% to cephalexin. Resistance to the quinolones was 42% to ciprofloxacin, 21% to norfloxacin, and 17% to nalidixic acid. Against the aminoglycosides, resistance was 14% to kanamycin and 12% to gentamicin.

Goat isolates showed resistance of 100%, 97%, 94%, 84%, and 75%, respectively, to erythromycin ampicillin, tetracycline, chloramphenicol, and trimethoprim/sulfamethoxazole. Against the cephalosporins, resistance was 100% to cephalexin and 91% to cefotaxime. Resistance to the quinolones was 69% to nalidixic acid, 62% to ciprofloxacin, and 47% to norfloxacin. Resistance to the aminoglycosides was 44% to kanamycin and 34% to gentamicin.

Pig isolates showed 100%, 97%, 86%, 60%, and 54% resistance, respectively, to erythromycin, ampicillin, chloramphenicol, tetracycline, and trimethoprim/sulfamethoxazole. Against the cephalosporins resistance was 100% to cephalexin and 97% to cefotaxime. Resistance to the quinolones was 51% to nalidixic acid, 35% to ciprofloxacin, and 22% to norfloxacin. Resistance to the aminoglycosides was 0% to kanamycin and 30% to gentamicin.

No resistance (0%) was recorded against imipenem; however intermediate susceptibility was observed among strains from all the animals (Table 6).

### 3.6. Species-Specific Resistance Profile of Animals

Resistance of* C. jejuni* strains to the quinolones was 23% to nalidixic acid, 25.5% to norfloxacin, and 40.2% to ciprofloxacin. Against the cephalosporins, resistance was 92.6% and 99.5% to cefotaxime and cephalexin, respectively. Resistance to aminoglycosides was 17.1% to kanamycin and 21.1% to gentamicin. Resistance to erythromycin, ampicillin, chloramphenicol, tetracycline, and trimethoprim/sulfamethoxazole, respectively, was 99.5%, 96.5%, 87.7%, 69.8%, and 58.8%.

Resistance among* C. coli* strains to the quinolones was 2.9% each to nalidixic acid and norfloxacin and 37.7% to ciprofloxacin. Resistance to the cephalosporins was 66.7% to cefotaxime and 72.5% to cephalexin. Against the aminoglycosides, resistance was 1.4% to gentamicin and 8.7% to kanamycin. Resistance to erythromycin, tetracycline, ampicillin, trimethoprim/sulfamethoxazole, and chloramphenicol, respectively, was 100%, 89.8%, 84%, 71%, and 43.5%.

Resistance of* C. lari* to the quinolones was 100% to nalidixic acid, 93.3% to ciprofloxacin, and 83.3% to norfloxacin. Against the cephalosporins, resistance was 93.3% to the cefotaxime and 100% to the cephalexin. Resistance to aminoglycosides was 26.7% to gentamicin and 36.7% to kanamycin. Resistance to erythromycin was 100%, to ampicillin and tetracycline 96.7% each, to trimethoprim/sulfamethoxazole 90%, and to chloramphenicol 66.7% (Table 7).

### 3.7. Multidrug Resistance (MDR) in* Campylobacter* Species from Animals

Resistance to three (3) or more antibiotics was defined as multidrug resistance (MDR) in this study. Strains of* C. jejuni*,* C. coli*, and* C. lari*, respectively, showed multidrug resistance of 66.6% (156/234), 20.5% (48/234), and 12.8% (30/234). Multidrug resistance in* C. jejuni* strains from the animal carcasses was consistently higher than faecal strains across studied animals. Contrastingly, higher MDR was observed in* C. coli* strains obtained from faecal sources of the animals. With the exception of sheep, the differences in MDR of faecal and carcass strains from pigs (*p* = 0.000), goats (*p* = 0.003), and cattle (*p* = 0.012) were statistically significant.

## 4. Discussion

The fastidious nature of* Campylobacter* coupled with its susceptibility to environmental stresses such as heat, drying, and exposure to air often results in damaged cells which hamper their recovery to a greater extent than most bacteria. Again, a viable but nonculturable (VBNC) state exhibited by* Campylobacter* can result in underestimation or nondetection of the organism by culture-based techniques, yet cells in this state can still infect susceptible hosts [10]. Notwithstanding,* Campylobacter* have been isolated from animals in different countries at varying rates. Documented range of 5–49% has been reported in sheep and goats [11, 12], 0–80% in cattle, and 50–100% in pigs [13, 14].

In our study,* Campylobacter* prevalence ranged from 13.2 to 28.7% in faecal samples and from 23.9 to 36.3% in the carcasses and again* Campylobacter* recoveries from carcasses were more than from faeces. This could be due to contamination of carcasses with intestinal contents during manual skinning, evisceration, carcass washing, and processing at the abattoir. Similarly, A Mpalang et al. [15] recorded 50%* Campylobacter* prevalence in carcasses compared to 20% in faecal samples. The 13.2% recovery rate from cattle faeces in this study compares to the 12.7% in Ethiopia but is lower than studies in USA (26.7%–29.1%), Finland (31.1%), and Canada (76–95%) [16–19].* Campylobacter* contamination in cattle carcasses are generally low; however, Noormohamed and Fakhr [20] reported 78% from beef livers which is higher than the 35.2% obtained in this study. Lower isolation rates have also been reported in different countries [18, 21].

The 18.6%* Campylobacter* prevalence in sheep faeces is lower than the 23% and 38.0% rates reported in studies in Ghana and Ethiopia, respectively [17, 22], but higher than the 4.5% reported in USA [23]. In sheep carcasses, rates of 11.0% have been reported in Ethiopia which is lower than the 35.9% obtained in this study although a higher rate of 72.2% has been described in Greece [24, 25]. The 18.5%* Campylobacter* recovery in goat faeces from this study was lower than the 33% and 20% reported by Abrahams et al. [22] and Salihu et al. [11] in studies in Ghana and Nigeria, but higher than the 3.2% reported in USA [15, 23]. Goat meat is not known to be a major source of campylobacteriosis; however, the high utilization of mutton in local Ghanaian dishes may contribute to the increased transmission source. In goat carcasses,* Campylobacter* contamination rates of 50% and 63.5% have been reported from Congo and Greece, with 9.4% in Ethiopia compared to the 23.9% in our study [15, 24, 25].

*Campylobacter* prevalence of 28.7% recovered from pig faeces was lower compared to 42.4%, 50%, and 71.4% in studies in Japan, Ethiopia, and Mexico [17, 26, 27]. Geographical distribution of* Campylobacter* contamination in pig carcasses is fairly low (2.0–25.3%) [20, 27] which is still lower than the 36.3% in this study.* Campylobacter* contamination in pig carcasses has been documented in various studies [28]. It must be noted that there were no significant differences in contamination rates of the various food animal carcasses which may be as a result of the levels of colonization of slaughter animals, abattoir hygiene, slaughter, and dressing methods [29].

*Campylobacter jejuni* and* C. coli* were the most commonly identified species although* C*.* coli *were more in faecal samples and* C. jejuni* were more in carcasses. Similar findings were made by A Mpalang et al. [15] who also recovered more* C. coli *(26.1%) than* C. jejuni *(10.1%) from faecal isolates of goats in Ethiopia; 25.9%* C. coli* and 3.4%* C. jejuni* in nondiarrhoeic goat faeces have been documented in South Africa [30]. These findings suggest that* C. coli* are more common in Africa [31].* Campylobacter jejuni* dominance in carcasses (83.9%) is comparable to work by Wieczorek et al. [4] and Noormohamed and Fakhr [20]. However, a number of studies have rather shown higher* C. jejuni* in faecal samples of animals [17, 23, 26].

Although* C. coli* is the most commonly identified species in pigs [32, 33]; an interesting pattern was discovered in our study where* C. jejuni* and* C. coli* isolations were similar (48.2% each) in pig faeces and a rather higher* C. jejuni* (75.6%) than* C. coli* (10.8%) in the carcasses. Matthew-Belmar et al. [34] and Kramer et al. [35] recorded more* C. jejuni* (53.5%) than* C. coli* (46.5%) from pigs in Grenada and UK, respectively. However, higher* C. coli* than* C. jejuni* have been recorded in Nigeria [36], in Ethiopia [17], and in Poland [21]. Other studies do show that* C. jejuni* may coexist with* C. coli* in pigs but usually the* C. jejuni* are always present in 10–100-fold lower numbers than* C. coli* [37]. In Ghana, most of the farms rear multiple animals and it could be that the pigs may have acquired the* C. jejuni* from poultry on the same farm [23, 38].

Conventional culture methods and API biochemical reactions of isolates from food animals were 95.8% in agreement with the results of PCR for identification and differentiation of* Campylobacter* species which is comparable with data from other studies [9, 39]. The bacteriological methods (culture and API) are as reliable as the molecular PCR (*IpxA* gene) method in detecting* Campylobacters *from animals.

The limitation in detecting antibiotic resistance in this study was as it is in all studies dependent on culture that the resistance rates were determined according to the bacteria species that were culturable at the time of analysis. Direct PCR on the specimens might be more sensitive but not detecting the actual species where the antibiotic resistance genes are present in.

High levels of resistance were expressed against most of the antibiotics. Faecal and carcass strains showed resistance range of 97–100% to erythromycin which is consistent with work in Nigeria and Spain, where resistance of 81–82.6% to erythromycin has been described [3, 5], but lower rates have been reported in Ethiopia (60.3%) and USA (55%) [6, 20].

Similarly, Ampicillin resistance ranged from 93% to 100% which agrees with studies in India [40] and in Ethiopia, Spain, and USA [3, 6, 20]. Resistance to the cephalosporins ranged from 62 to 97% to cefotaxime and 72 to 100% to cephalexin which is consistent with reports from Ghana (95.8%) and other countries where rates of 95.8–100% have been established [6, 20, 22, 40]. Resistance range of 6–69% was observed in our study against the quinolones which is lower than rates described in USA (100%), Ethiopia (80.5%), Poland (86.8%–88.1%), and Thailand (91–100%) [4, 6, 20, 41] and for the aminoglycosides a range of 0–44% was recorded which is comparable to rates from Poland, Grenada, and Spain [3, 4, 34] but lower than established rates in Nigeria and Ethiopia [5, 6].

Resistance to tetracycline was between 58 and 94% which is consistent with documented rates in Poland, USA, Ethiopia, and Thailand [4, 6, 20, 41] as resistance to chloramphenicol was in the range of 45–88% higher than rates of 61.5% and 67.4% reported against chloramphenicol in Ethiopia and Nigeria, respectively [5, 6]. No resistance was observed against imipenem although intermediate susceptibility was found in both faecal and carcass isolates. Generally, resistant strains were commonly found in cattle and sheep compared to goats and pigs and resistance was also higher in the carcasses than in the faecal isolates.

The extensive application of antibiotics in animal husbandry for therapy, prophylaxis, and growth promotion has often been associated with the spread of resistance. Another factor contributing to the increase and spread of resistance is intensive rearing which promotes clinical infections in animals leading to widespread prophylactic usage of drugs which may be unwarranted. Currently in Ghana livestock production is changing from free range to commercial productions and may have added to the high level resistance currently documented.

Antibiotic use in animal feed as growth promotants also plays a significant role in the spread of resistance. Worldwide, antibiotics are widely used in livestock and poultry for growth promotion to enhance feed utilization and production [14]. In Ghana, 98% of livestock farmers use antibiotics on their farms as growth promoters and in the management of diseases. The antibiotics used are mainly tetracyclines (oxytetracycline, doxycycline, remacycline, and chlortetracycline), sulphadimidine, dihydrostreptomycin, piperazine, albendazole, tylosin, ivermectin, and benzylpenicillin which can lead to possible cross- and co-resistance [42, 43]. In a study in Kumasi, the knowledge of livestock farmers on antibiotics, withdrawal periods, and dosages was very low and farmers usually depended more on fellow farmers than veterinarians for antibiotic knowledge. Poor dosing practices especially when an antibiotic failed to resolve an infection were a common practice. In such cases, different antibiotics were tried and abused until the disease was treated [43].

Also the challenge of distinguishing different antibiotic brands of the same active ingredient resulted in the application of different antibiotic brands of the same active ingredient which saw no improvement in the disease condition [43]. In the Northern Region of Ghana, Addah et al. [44] reported of nonadherence to dosing and withdrawal periods among several livestock farmers. These practices ultimately increase antibiotic residues in faecal content and carcasses of these animals which is evident in the high level resistance established in this study.

## 5. Conclusion

The study has revealed multidrug resistant* Campylobacter* species in the faecal content and carcasses of healthy livestock animals in Ghana indicating possible risks of infection to people through consumption of contaminated animal products or by direct contact with animals. Moreover high levels of resistance observed among the* Campylobacter* species to the common and cheap antibiotics raise uncertainties about their effectiveness in the treatment of animal and human diseases in the study region. It is urgent that extensive education and training are given to livestock farmers on judicious application of antibiotics and a national antibiotic resistance management team setup to monitor and control antibiotic use in both human and veterinary medicine.

## Figures and Tables

**Table 1:** PCR primers of *IpxA *gene of *Campylobacter* species used in this study.

Primers	Sequence (5′-3′)	Size (bp)
*IpxA C. coli* (F)	AGACAAATAAGAGAGAATCAG	391
*IpxA C. jejuni* (F)	ACAACTTGGTGACGATGTTGTA	331
*IpxA C. lari* (F)	TRCCAAATGTTAAAATAGGCGA	233
*IpxA C. upsaliensis* (F)	AAGTCGTATATTTTCYTACGCTTGTGTG	206
*IpxARKK2m* (R)	CAATCATGDGCDATATGASAATAHGCCAT	

F = forward; R = reverse.

**Table 2:** Isolation rate of *Campylobacter *from faeces and carcasses of animals.

Animal	Total number of samples	Number of *Campylobacter* spp. identified *N* (%)
	Faeces	
Cattle	121	16 (13.2)
Sheep	118	22 (18.6)
Goat	135	25 (18.5)
Pig	101	29 (28.7)

	Carcass	
Cattle	110	38 (34.5)
Sheep	117	42 (35.9)
Goat	134	32 (23.9)
Pig	102	37 (36.3)

**Table 3:** Association between isolation rate of faecal and carcass samples of animals.

Source	*Campylobacter* occurrence		*p *value
	Faeces	Carcass	
	*n* (%)	*n* (%)	
Cattle	16 (13.2)	38 (34.5)	<0.001
Sheep	22 (18.6)	42 (35.9)	0.003
Goat	25 (18.5)	32 (23.9)	0.268
Pig	29 (28.7)	37 (36.3)	0.250

**Table 4:** Distribution of *Campylobacter* species in the carcasses and faecal contents of animals.

Source	Number of samples	*C. jejuni*	*C. doylei*	*C. coli*	*C. lari*
		*n* (%)	* n* (%)	*n* (%)	*n* (%)
Cattle faeces	16	4 (25.0)	3 (18.7)	7 (43.7)	2 (12.5)
Cattle carcass	38	32 (84.2)	0 (0)	5 (13.1)	1 (2.6)
Sheep faeces	22	6 (27.2)	1 (4.5)	9 (40.9)	6 (27.2)
Sheep carcass	42	39 (92.8)	2 (4.7)	0 (0.0)	1 (2.3)
Goat faeces	25	9 (36.0)	0 (0)	14 (56.0)	2 (8.0)
Goat carcass	32	26 (81.3)	0 (0)	0 (0)	6 (18.7)
Pig faeces	29	14 (48.2)	0 (0)	14 (48.2)	1 (3.4)
Pig carcass	37	28 (28.4)	1 (2.7)	4 (10.8)	4 (10.8)

C. *doylei *** = **C. *jejuni* subsp. *doylei*.

**Table 5:** Susceptibility patterns of faecal isolates of animals.

Antibiotic	Animal											
	Cattle (*N* = 16)			Sheep (*N* = 22)			Goat (*N* = 25)			Pig (*N* = 29)		
	S	I	R	S	I	R	S	I	R	S	I	R
Nalidixic acid	50	NA	50	64	NA	36	92	NA	8	90	NA	10
Tetracycline	13	6	81	9	0	91	12	12	76	7	7	86
Erythromycin	0	NA	100	0	NA	100	0	NA	100	0	NA	100
Ciprofloxacin	37	19	44	14	36	50	48	24	28	41	35	24
Chloramphenicol	6	6	88	4	41	55	12	24	64	14	41	45
Ampicillin	0	19	81	4	5	91	8	4	88	3	14	83
Cefotaxime	25	0	75	9	5	86	28	4	68	14	24	62
Kanamycin	50	44	6	27	55	18	68	24	8	38	52	10
Gentamicin	62	38	0	68	18	14	80	20	0	86	4	10
Norfloxacin	50	31	19	59	5	36	76	12	12	76	7	17
Trimethoprim/sulfamethoxazole	44	0	56	14	0	86	40	0	60	52	0	48
Cephalexin	19	NA	81	9	NA	91	28	NA	72	14	NA	86
Imipenem	69	31	0	68	32	0	92	8	0	72	28	0

S = sensitive; I = intermediate; R = resistant; NA = intermediate not available, values presented in percentages.

**Table 6:** Susceptibility pattern of carcass isolates of animals.

Antibiotic	Animal											
	Cattle (*N* = 38)			Sheep (*N* = 42)			Goat (*N* = 32)			Pig (*N* = 37)		
	S	I	R	S	I	R	S	I	R	S	I	R
Nalidixic acid	87	NA	13	83	NA	17	31	NA	69	49	NA	51
Tetracycline	24	18	58	40	12	48	6	0	94	24	16	60
Erythromycin	3	0	97	0	NA	100	0	NA	100	0	NA	100
Ciprofloxacin	34	24	42	38	38	24	19	19	62	49	16	35
Chloramphenicol	5	8	87	3	14	83	6	10	84	3	11	86
Ampicillin	0	3	97	5	2	93	0	3	97	3	0	97
Cefotaxime	8	0	92	0	0	100	9	0	91	0	3	97
Kanamycin	68	13	19	57	29	14	19	37	44	57	43	0
Gentamicin	79	3	18	88	0	12	60	6	34	62	8	30
Norfloxacin	68	11	21	83	10	7	37	16	47	65	13	22
Trimethoprim/sulfamethoxazole	53	5	42	48	0	52	25	0	75	46	0	54
Cephalexin	3	NA	97	2	NA	98	0	NA	100	0	NA	100
Imipenem	76	24	0	81	19	0	84	16	0	76	24	0

S = sensitive; I = intermediate; R = resistant; NA = intermediate not available, values presented in percentages.

**Table 7:** Species-specific resistance profile of strains from food animals.

	*C. jejuni *= 204	*C. coli *= 69	*C. lari = *30
Antibiotic	% resistance	% resistance	% resistance
Nalidixic acid	23	2.9	100
Cefotaxime	92.6	66.7	93.3
Erythromycin	99.5	100	100
Tetracycline	69.8	89.8	96.7
Kanamycin	17.1	8.7	36.7
Gentamicin	21.1	1.4	26.7
Ampicillin	96.5	84	96.7
Imipenem	0	0	0
Cephalexin	99.5	72.5	100
Ciprofloxacin	40.2	37.7	93.3
Chloramphenicol	87.7	43.5	66.7
Norfloxacin	25.5	2.9	83.3
SXT	58.8	71	90
